# Physical Activity and Self-Reported Academic Achievement Among Rural Left-Behind Children: The Serial Mediating Roles of Character Strengths and Self-Control

**DOI:** 10.3390/bs16081345

**Published:** 2026-08-05

**Authors:** Wei Xiang, Siyi Wang, Xiaobing Luo

**Affiliations:** School of Physical Education and Sports, Central China Normal University, Wuhan 430079, China

**Keywords:** rural left-behind children, physical activity, self-reported academic achievement, character strengths, self-control, serial mediation

## Abstract

Physical activity may be related to self-reported academic achievement among rural left-behind children, but the psychological processes associated with this relationship remain unclear. This cross-sectional study examined the association between physical activity and self-reported academic achievement and tested the indirect roles of character strengths and self-control. A total of 450 rural left-behind children completed the Physical Activity Rating Scale, the Character Strengths Scale for Chinese Elementary and Middle Schools, the Academic Achievement Self-Assessment Scale, and the Dual-System Scale for Adolescent Self-Control. Pearson correlation and serial mediation analyses were conducted. Physical activity, character strengths, self-control, and self-reported academic achievement were positively correlated. Physical activity was positively associated with self-reported academic achievement, and the indirect effects through character strengths and self-control were significant both separately and sequentially. These findings suggest that character strengths and self-control may help explain the association between physical activity and self-reported academic achievement. Because the data were cross-sectional and self-reported, causal and temporal conclusions cannot be drawn.

## 1. Introduction

Rural left-behind children are a population that has emerged during China’s socioeconomic development. They are generally defined as children under 16 years of age with rural household registration who cannot live with both parents because both parents have migrated for work, or because one parent has migrated and the other is unable to provide adequate care. According to China’s Child Population Situation in 2020: Facts and Figures, 41.77 million rural left-behind children lived in China in 2020. Long-term separation from parents may limit the educational support and daily care available to these children, and their academic achievement is often lower than that of non-left-behind children (Hu et al., 2020). Parental return alone may not immediately eliminate this academic disadvantage (Zheng & Wu, 2014). Academic achievement is also associated with the physical and psychological adjustment of rural left-behind children. Higher academic achievement may protect against depressive symptoms (Guang et al., 2017), whereas lower achievement has been associated with a greater likelihood of involvement in school bullying (Feng et al., 2020). It is therefore important to examine factors associated with academic achievement in this population and the psychological pathways that may underlie these associations.

### 1.1. The Association Between Physical Activity and Academic Achievement Among Rural Left-Behind Children

A common concern is that time spent in physical activity may reduce study time and impair achievement in academic subjects. This concern may also lead schools to reduce physical education time in favor of classroom instruction. However, meta-analytic evidence indicates that physical activity does not impair academic achievement and may support it (Watson et al., 2017; D. Li et al., 2023). Several pathways may account for this relationship. First, physical fitness provides a foundation for sustained learning and has been positively associated with academic achievement (García-Hermoso et al., 2021; de Bruijn et al., 2018). Second, physical activity may support attention, cognitive flexibility, and working memory in children and adolescents (de Greeff et al., 2018; Haverkamp et al., 2020). Third, physical activity may reduce stress and anxiety, promote positive emotions, and improve life satisfaction (Rodriguez-Ayllon et al., 2019), all of which are relevant to effective learning. These pathways may also be relevant to rural left-behind children. In addition, physical activity is generally accessible and requires fewer material resources than many forms of supplementary education, which may make it particularly relevant for children in resource-constrained rural settings. Accordingly, this study proposed the following hypothesis:

**Hypothesis** **1.** *Physical activity is positively associated with self-reported academic achievement among rural left-behind children.*

### 1.2. The Indirect Role of Character Strengths

Character strengths are positive traits expressed through thoughts, feelings, and behaviors. Peterson and Seligman (2004) organized these traits into 24 strengths under six core virtues. Based on this framework, Guan et al. (2009) developed a scale for Chinese primary and secondary school students that includes 15 character strengths across six dimensions, such as bravery, perseverance, kindness, tolerance, and teamwork. Character strengths have been associated with school grades (Rahe & Jansen, 2025) and may provide psychological resources that help students respond constructively to academic difficulties. For example, character strengths have been associated with well-being in young people (Park & Peterson, 2006a) and show longitudinal associations with academic self-efficacy (Datu & Mateo, 2020). These resources may be particularly relevant to rural left-behind children, who often face persistent family and environmental stressors. Sports and other structured physical activities may provide opportunities to practise persistence, cooperation, fairness, and rule-following. On this basis, the following hypothesis was proposed:

**Hypothesis** **2.** *Character strengths show a significant indirect effect in the association between physical activity and self-reported academic achievement among rural left-behind children.*

### 1.3. The Indirect Role of Self-Control

Self-control refers to the ability to regulate immediate impulses and behavior in pursuit of longer-term goals. It is closely related to learning because students must often persist with important academic tasks despite competing preferences or distractions (Duckworth et al., 2019). In experimental choice settings, students with higher self-control spend more time on demanding academic tasks rather than immediately rewarding alternatives (Galla et al., 2014). Self-control has also been associated with academic achievement (Troll et al., 2021) and educational attainment (Moffitt et al., 2011). This ability may be especially relevant when parental supervision is limited. Physical activity and self-control have been positively associated among adolescents (Boat et al., 2024). Self-control models also suggest that regulatory effort can have short-term after-effects on subsequent emotional and behavioral responses (Kelley et al., 2019). Structured physical activity requires goal-directed effort, persistence, and adherence to rules, which may provide opportunities to practise self-regulation. Accordingly, this study proposed the following hypothesis:

**Hypothesis** **3.** *Self-control shows a significant indirect effect in the association between physical activity and self-reported academic achievement among rural left-behind children.*

### 1.4. The Serial Indirect Roles of Character Strengths and Self-Control

**Figure 1:**
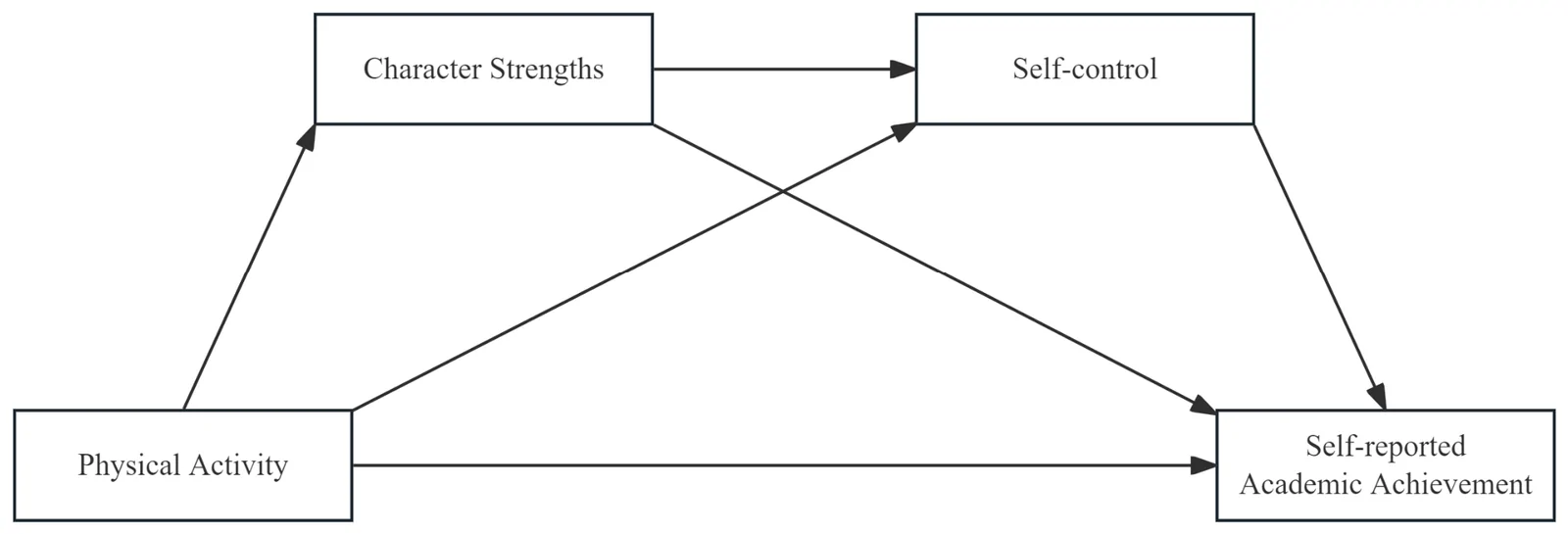
Hypothesized serial mediation model linking physical activity, character strengths, self-control, and self-reported academic achievement.

Character strengths and self-control are closely related psychological resources. Character strengths may reduce negative emotions and support subjective well-being (Kor et al., 2019). Negative emotions can weaken self-control by consuming regulatory resources (Shmueli & Prochaska, 2012; Chester et al., 2016), whereas positive emotions may support their preservation and recovery. Character strengths may also strengthen the motivation to follow personal principles and pursue long-term goals. For example, kindness and tolerance have been associated with greater self-control (H. Y. Zhang et al., 2021; Inez et al., 2020). Taken together, physical activity may be associated with stronger character strengths, which may in turn be associated with greater self-control and higher self-reported academic achievement. The following serial mediation hypothesis was therefore proposed:

**Hypothesis** **4.** *Character strengths and self-control show a significant serial indirect effect in the association between physical activity and self-reported academic achievement among rural left-behind children (see Figure 1).*

### 1.5. Originality and Novel Contributions of the Present Study

Scholars have extensively probed potential psychological pathways that may correlate physical activity with academic achievement in children and adolescents, among which the cognitive function pathway enjoys the strongest empirical documentation. Previous studies have reported positive associations among physical activity, cognitive functioning, and academic achievement (Álvarez-Bueno et al., 2016; Z. Zhang et al., 2026). As relevant research advances, an increasing number of researchers have moved beyond cognitive dimensions to examine the mediating roles of other psychological variables in this association. Systematic reviews and meta-analyses have identified a suite of mediators linking physical activity to academic achievement, including cognitive self-regulation, self-esteem, self-image, self-efficacy, and stress (Visier-Alfonso et al., 2022; Zarazaga-Peláez et al., 2024). Nevertheless, existing literature has rarely investigated the potential mediating pathway of character strengths in the relationship between physical activity and academic achievement among children and adolescents. Character strengths constitute a core operational construct in positive psychology for quantifying positive personality traits, and prior research has documented their significant correlations with individuals’ well-being and achievement (Park & Peterson, 2008; Hausler et al., 2017). By testing the mediating effect of character strengths in the association between physical activity and adolescents’ academic achievement, the present study addresses theoretical gaps in extant research and enriches empirical evidence for the field of motor development from a positive psychology perspective. From an educational practice standpoint, character strengths represent relatively stable personality traits, conferring more sustained value compared with volatile psychological indicators such as self-efficacy and stress. The findings identify character strengths and self-control as candidate variables for future longitudinal and intervention research within school-based education. Furthermore, this study examines the serial mediating pathway of “character strengths–self-control”. This analysis corroborates the sequential statistical associations of stable positive personality traits like character strengths on other psychological variables. Additionally, it offers a novel interpretative framework rooted in personality psychology to unpack the potential psychological processes underlying the link between physical activity and adolescents’ academic achievement.

## 2. Materials and Methods

### 2.1. Participants and Procedure

By definition, left-behind children refer to those under 16 years of age with rural household registration who cannot live with both parents because both parents have migrated for work, or because one parent has migrated and the other is unable to provide adequate care. In accordance with the school-age system for children and adolescents in China, students graduate from junior high school at age 15, meaning all primary and junior high school students are under 16. Therefore, this study recruited rural left-behind children attending primary and junior high schools as research participants. A multistage sampling procedure was used. Eight primary and secondary schools with relatively large populations of rural left-behind children were purposively selected from three townships in Yichang City, Hubei Province. Within the selected schools, eligible students in Grades 5–9 were recruited by class-based cluster random sampling. Individuals presenting motor impairments or psychological disorders, those whose place of origin/residence was not rural, as well as those who failed to meet the inclusion criteria for left-behind children, were excluded at the initial screening stage. School administrators and classroom teachers granted permission for data collection, and written informed consent was obtained from the participants (the left-behind children themselves) and their legal guardians before the survey.

Questionnaires were distributed by trained research staff during supervised study periods. After classroom teachers introduced the research staff to the students, they left the classroom and did not oversee the questionnaire completion process to reduce perceived pressure and social response bias. Participants were informed that participation was fully voluntary: they could decline to take part or stop filling out the questionnaire at any time without penalty, and non-participation would have no impact on their academic achievement or daily school life. All responses were anonymous, as research staff did not collect or access students’ personal identifying information. Each questionnaire took approximately 15 min to complete and was collected immediately upon completion.

A total of 460 questionnaires were distributed and returned. After questionnaires with substantial missing data, incomplete responses, or invariant response patterns were excluded, 450 valid questionnaires remained, yielding a valid response rate of 97.8%. Participant characteristics are presented in Table 1.

### 2.2. Research Tools

#### 2.2.1. Physical Activity Rating Scale

The Chinese version of the Physical Activity Rating Scale revised by Liang (1994) was used to assess physical activity. The scale contains three items measuring exercise intensity, duration, and frequency, each rated on a 5-point scale. A composite physical activity score was calculated as intensity × (duration − 1) × frequency, with higher scores indicating greater physical activity. The scale has been widely used in questionnaire-based studies in China (Zuo et al., 2025; Y. Lin et al., 2025). Different from conventional reflective scales, this instrument generates a multiplicative composite integrating three separate behavioral components. Accordingly, Cronbach’s alpha has limited applicability for evaluating the reliability of this index and is reported herein only for comparison with previous studies. The Cronbach’s alpha was 0.67 in the present sample. It should be noted that the observed Cronbach’s alpha of 0.67 arises mainly because physical activity level can only be fully captured by combining intensity, duration and frequency, yet these three indicators are conceptually independent and tend to exhibit low pairwise correlations. Similar alpha values between 0.6 and 0.7 have been widely documented in previous research employing the same multiplicative scoring method for this scale (Zuo et al., 2025; Y. Lin et al., 2025).

#### 2.2.2. Academic Achievement Self-Assessment Scale

The Ministry of Education of the People’s Republic of China stipulates that compulsory education schools shall adopt grade-based evaluation for routine examinations. Examination results shall not be publicly released and shall only be communicated to students and parents through appropriate channels to resolutely overcome the score-only mindset. For this reason, schools could not provide objective original exam scores. Nevertheless, students were clearly aware of their personal achievement grades, so self-reported academic achievement was measured using the Academic Achievement Self-Assessment Scale developed by Wigfield et al. (1991). The seven items ask students to rate their overall achievement and their relative standing in Chinese, mathematics, and a foreign language. The measure therefore reflects students’ perceived academic achievement rather than officially recorded examination scores. Cronbach’s alpha was 0.88 in the present sample.

#### 2.2.3. Character Strengths Scale for Chinese Elementary and Middle School Students

The Character Strengths Scale for Chinese Elementary and Middle School Students was adapted for Chinese students by Guan et al. (2009) from the VIA-Youth framework developed by Park and Peterson (2006b). The scale contains 61 items assessing 15 character strengths across six dimensions: cognition, bravery, humanity, fairness, temperance, and transcendence. Cronbach’s alpha was 0.95 in the present sample.

#### 2.2.4. Dual-System Scale for Adolescent Self-Control

The Chinese version of the Dual-System Scale for Adolescent Self-Control, developed by Dvorak and Simons (2009) and revised by Xie et al. (2014), was used to assess self-control. The 21-item scale comprises control and impulsivity systems and includes five factors: impulsivity, distractibility, low delay of gratification, problem solving, and future time perspective. Cronbach’s alpha was 0.89 in the present sample.

### 2.3. Statistical Analysis

Data were analyzed using IBM SPSS Statistics 26.0 for Windows. Pearson correlation analysis was used to examine associations among physical activity, character strengths, self-control, and self-reported academic achievement. Serial mediation analysis was conducted using the PROCESS macro version 4.3.1 for SPSS. Grade, gender, left-behind type, and only-child status were included as covariates. Indirect effects were estimated using 5000 bootstrap samples and 95% bias-corrected confidence intervals. An indirect effect was considered statistically significant when its confidence interval did not include zero.

## 3. Results

### 3.1. Assessment of Common Method Bias

All core variables were measured using self-report questionnaires, which may introduce common method variance. Harman’s single-factor test was therefore conducted. Exploratory factor analysis of all questionnaire items identified 25 factors with eigenvalues greater than 1. The first factor explained 22.18% of the total variance, below the conventional 40% threshold. This result suggests that no single factor dominated the observed variance; however, Harman’s test alone cannot rule out common method variance. Beyond Harman’s single-factor test, we further assessed common method bias via the full collinearity VIF approach proposed by Kock (2015). The results show that the full collinearity VIF values of physical activity, self-reported academic achievement, self-control and character strengths were 1.158, 1.329, 1.523 and 1.466, respectively, all below the critical threshold of 3.3. Harman’s single-factor test and full collinearity VIF analysis did not identify a dominant common method factor in this study. Nevertheless, given that all core variables were concurrently measured via self-report questionnaires, common method variance cannot be fully ruled out and may still exert subtle influences on the study findings.

### 3.2. Descriptive Statistics and Correlations

Physical activity, character strengths, self-control, and self-reported academic achievement were significantly and positively correlated with one another (Table 2).

### 3.3. Serial Mediation Analysis

Given the significant correlations among the study variables, a serial mediation model was estimated using the PROCESS macro. Grade, gender, left-behind type (dummy coded, with rural left-behind children with only father migrating out set as the reference group), and only-child status were entered as covariates. Indirect effects were assessed using 5000 bootstrap samples and 95% bias-corrected confidence intervals.

Physical activity was positively associated with self-reported academic achievement (β = 0.327, t = 7.525, *p* < 0.001). When physical activity, character strengths, and self-control were entered simultaneously, the association between physical activity and self-reported academic achievement remained significant (β = 0.210, t = 4.995, *p* < 0.001). Physical activity was positively associated with character strengths (β = 0.245, t = 5.389, *p* < 0.001) and self-control (β = 0.175, t = 4.221, *p* < 0.001). Character strengths were positively associated with self-control (β = 0.491, t = 12.332, *p* < 0.001) and self-reported academic achievement (β = 0.207, t = 4.305, *p* < 0.001). Self-control was also positively associated with self-reported academic achievement (β = 0.225, t = 4.528, *p* < 0.001) (Table 3).

**Figure 2:**
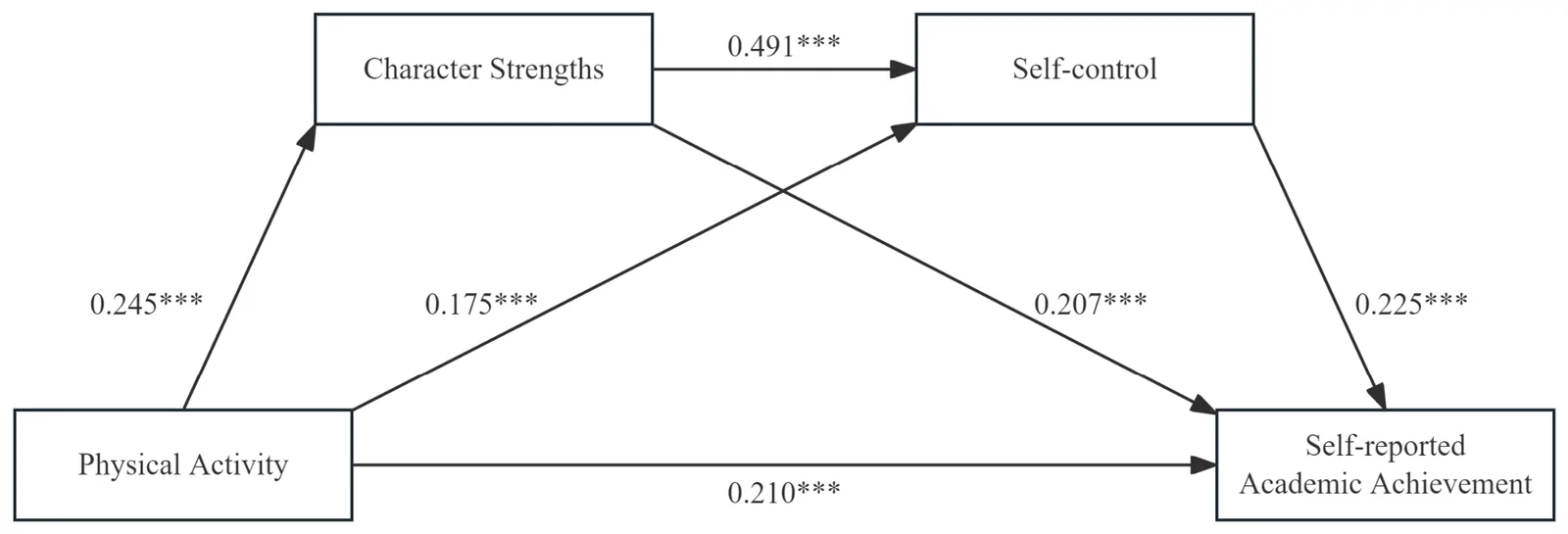
Standardized coefficients for the serial mediation model. *** *p* < 0.001. **Note:** an indirect effect was considered statistically significant when the 95% bootstrap confidence interval did not include zero; all coefficients are standardized.

Bias-corrected bootstrap analysis indicated that the total indirect effect was statistically significant (effect = 0.117, 95% bootstrap CI [0.079, 0.160]). The indirect effect through character strengths was significant (effect = 0.051, 95% bootstrap CI [0.022, 0.085]), as was the indirect effect through self-control (effect = 0.039, 95% bootstrap CI [0.015, 0.070]). The serial indirect effect through character strengths and self-control was also significant (effect = 0.027, 95% bootstrap CI [0.012, 0.045]) (Table 4). All direct, indirect, total effects and proportion mediated values were calculated based on standardized coefficients with the same metric scale. Notably, the proportion mediated reflects a statistical decomposition of the observed association rather than a causal proportion. The full serial mediation model is presented in Figure 2.

From an educational perspective, the magnitude of these indirect effects reflects the relative correlational weight of each psychological pathway linking physical activity and self-reported academic achievement. The total indirect effect of 0.117 aligns with a modest overlapping correlational pattern of these psychological pathways in the observed cross-sectional association between physical activity and self-reported academic achievement. Among separate parallel pathways, the indirect pathway involving character strengths exhibits a larger effect size (0.051) relative to the pathway centering on self-control (0.039), suggesting character strengths may have a stronger concurrent correlational linkage with self-reported academic achievement within this psychological system. The smaller serial indirect effect (0.027) further points to a sequential correlational pattern: individual differences in character strengths tend to co-occur with variations in self-control, and together they correspond to a modest additional indirect association with self-reported academic achievement. Taken together, these effect sizes reflect that psychological factors are meaningful correlates when exploring the connection between physical activity and self-reported academic achievement. These factors may be useful candidates for future longitudinal or intervention research to inform targeted educational support strategies for adolescents.

## 4. Discussion

This study examined the association between physical activity and self-reported academic achievement among rural left-behind children and tested the indirect roles of character strengths and self-control. Physical activity was positively associated with self-reported academic achievement. In addition, the indirect effects through character strengths and self-control were significant both separately and sequentially.

### 4.1. Physical Activity and Self-Reported Academic Achievement

The positive association between physical activity and self-reported academic achievement is consistent with previous research. Meta-analytic evidence suggests that acute physical activity is associated with better attention, whereas regular physical activity is associated with executive function and academic achievement (de Greeff et al., 2018). Physical fitness, particularly cardiorespiratory fitness, has also been associated with academic achievement in children and adolescents (Jaekel, 2024). Psychological factors such as self-efficacy may co-occur with the association between physical activity and academic achievement (Zarazaga-Peláez et al., 2024). The present findings extend this literature by examining character strengths and self-control as two related psychological factors. For rural left-behind children, physical activity may be especially relevant because it is relatively accessible and low-cost in comparison with many forms of supplementary education. Previous research has also suggested that adolescents from lower-income families may obtain comparatively greater academic and cognitive benefits from physical activity (Fang, 2020). Nevertheless, the present cross-sectional findings indicate association rather than causation.

### 4.2. The Indirect Role of Character Strengths

The first significant indirect effect operated through character strengths. Children who reported more physical activity also reported stronger character strengths, which were in turn associated with higher self-reported academic achievement. Physical activity is not limited to physical conditioning; sports and exercise can also provide opportunities to practise perseverance, cooperation, fairness, and respect for rules. These qualities correspond to several dimensions of character strengths (Huang, 2007). Character strengths may be particularly relevant to rural left-behind children because persistent stress, loneliness, and difficulties in psychological adjustment can reduce the personal resources available for coping with academic demands (Xiong et al., 2024; Y. Wang et al., 2021). From a stress and resource perspective, such pressures may weaken adaptation by depleting psychological resources (Kwag et al., 2011; Fan & Fan, 2021). Character strengths may support more positive academic emotions, lower academic frustration, and more constructive coping with academic challenges (M. Lin et al., 2018; Q. Li et al., 2019; W. Zhang & Wang, 2017). However, the cross-sectional design does not establish whether physical activity precedes character strengths or whether children with stronger character strengths are more likely to participate in physical activity. Longitudinal and intervention studies are needed to clarify temporal ordering.

### 4.3. The Indirect Role of Self-Control

The second significant indirect effect operated through self-control. Children who reported more physical activity also reported greater self-control, which was associated with higher self-reported academic achievement. Self-control may be especially important when daily parental supervision is limited because it supports persistence, resistance to distraction, and effective study behavior. From the perspective of self-control resource models, positive emotional experiences may help restore regulatory resources after effortful tasks (Baumeister et al., 2007; Shen et al., 2021). Physical activity has been associated with fewer negative emotions (T. Wang et al., 2024) and with positive experiences such as flow (Rodrigues et al., 2025). Structured physical activity may also provide repeated opportunities to practise persistence, behavioral regulation, and adherence to rules. These features offer a plausible explanation for the observed indirect effect. At the same time, the association may be bidirectional: children with greater self-control may also be more likely to maintain regular physical activity. The current cross-sectional design cannot determine the temporal direction of this relationship.

### 4.4. The Serial Indirect Roles of Character Strengths and Self-Control

The serial indirect effect through character strengths and self-control was statistically significant. One possible interpretation is that physical activity provides contexts in which children can practise positive personal qualities, and these qualities may support self-control through more positive emotions, stronger learning interest, and greater motivation (Weber et al., 2016; Shen et al., 2021). Motivation may also help individuals maintain task performance when self-regulatory resources are reduced (Gregersen et al., 2017). In this way, character strengths and self-control may jointly help explain why physical activity is associated with higher self-reported academic achievement among rural left-behind children. This interpretation is consistent with the proposed model, but the statistical sequence should not be treated as evidence of a confirmed causal mechanism. Longitudinal and experimental studies are needed to test whether changes in physical activity are followed by changes in character strengths, self-control, and self-reported academic achievement.

Left-behind children are not unique to China and are also found in other countries affected by labor migration (Antia et al., 2020). However, much of the literature on physical activity and academic achievement in this population has focused on China. Academic achievement is particularly important for children living with limited family and educational resources because it may shape their later educational and social opportunities. The present study therefore provides evidence from a population that remains underrepresented in the international literature.

This cross-sectional study identified direct and indirect statistical associations between physical activity and self-reported academic achievement. Accordingly, accessible physical activity may have merit within comprehensive support programmes for rural left-behind children. At the policy level, schools and local communities may consider ensuring adequate time, facilities, and equipment for regular physical activity. At the school level, after-school physical activity programs can be designed to be safe, inclusive, and developmentally appropriate. Physical education teachers may also use structured activities that encourage persistence, cooperation, rule-following, and self-regulation. The educational effects of such programs should be evaluated in longitudinal and intervention studies before firm causal recommendations are made.

## 5. Limitations

Several limitations should be considered when interpreting the findings.

(1) The cross-sectional design does not establish the temporal ordering or direction of the associations. Longitudinal studies and randomized controlled interventions are needed to examine whether changes in physical activity precede changes in character strengths, self-control, and self-reported academic achievement.
(2) Academic achievement was assessed using a self-report scale because official examination scores were not accessible. The measure may therefore reflect perceived academic competence and relative standing rather than students’ actual recorded achievement.
(3) Physical activity was assessed using a brief composite measure of intensity, duration, and frequency. Other dimensions, including activity type, setting, and organizational form, were not assessed and may show different associations with character strengths, self-control, and self-reported academic achievement.
(4) Participants were recruited from schools in one area of Hubei Province, which may limit the generalizability of the findings to rural left-behind children in other regions or countries. Future research could adopt multi-region sampling including rural left-behind children from multiple regions to further validate the robustness of the present conclusions.

## 6. Conclusions

Physical activity was positively associated with self-reported academic achievement among rural left-behind children. Character strengths and self-control showed significant indirect effects, both separately and sequentially, in this association. The findings indicate that character strengths and self-control are significant correlates within the association between physical activity and self-reported academic achievement. Because the study was cross-sectional and all core variables were self-reported, the findings should not be interpreted as evidence of causal or temporal relationships.

## Figures and Tables

**Table 1:** Participant characteristics.

Demographic Variable	Category	*n*	%
Gender	Male	218	48.4%
	Female	232	51.6%
Grade	Grade 5	57	12.7%
	Grade 6	100	22.2%
	Grade 7	85	18.9%
	Grade 8	92	20.4%
	Grade 9	116	25.8%
Whether being an only child	Yes	204	45.3%
	No	246	54.7%
Left-behind type	Father migrating out	25	5.6%
	Mother migrating out	166	36.9%
	Both parents migrating out	259	57.6%

**Table 2:** Means, standard deviations, and correlations among the study variables.

Variable	Mean	SD	1	2	3	4
1. Physical activity	25.95	22.54	1			
2. Self-reported Academic achievement	3.21	0.783	0.326 **	1		
3. Character strengths	3.46	0.58	0.252 **	0.383 **	1	
4. Self-control	3.63	0.63	0.279 **	0.416 **	0.531 **	1

**Note:** ** *p* < 0.01.

**Table 3:** Regression estimates for the serial mediation model.

Variable	Model 1: Self-Reported Academic Achievement			Model 2: Character Strengths			Model 3: Self-Control			Model 4: Self-Reported Academic Achievement		
	β	t	95% CI	β	t	95% CI	β	t	95% CI	β	t	95% CI
Grade	−0.128	−2.901 **	[−0.155, −0.036]	0.029	0.636	[−0.032, 0.091]	−0.109	−2.812 **	[−0.129, −0.035]	−0.113	−2.742 **	[−0.135, −0.024]
Gender	0.080	1.685	[−0.025, 0.324]	−0.017	−0.365	[−0.212, 0.151]	0.132	3.376 **	[0.103, 0.409]	0.055	1.332	[−0.061, 0.265]
Left-behind type	−0.096	−2.165 *	[−0.305, −0.017]	−0.052	−1.122	[−0.236, 0.065]	−0.027	−0.687	[−0.173, 0.08]	−0.073	−1.786	[−0.257, 0.01]
Only-child status	0.000	−0.010	[−0.177, 0.171]	−0.001	−0.012	[−0.181, 0.181]	−0.011	−0.294	[−0.177, 0.128]	0.002	0.057	[−0.158, 0.163]
Physical activity	0.327	7.525 ***	[0.248, 0.422]	0.245	5.389 ***	[0.158, 0.341]	0.175	4.221 ***	[0.092, 0.25]	0.210	4.995 ***	[0.132, 0.302]
Character strengths							0.491	12.332 ***	[0.416, 0.573]	0.207	4.305 ***	[0.114, 0.306]
Self-control										0.225	4.528 ***	[0.124, 0.32]
R^2^	0.136			0.067			0.332			0.267		
F	13.978 ***			6.395 ***			36.750			22.988 ***		

**Note:** * *p* < 0.05, ** *p* < 0.01, *** *p* < 0.001; all coefficients are standardized.

**Table 4:** Bootstrap estimates of the direct, total, and indirect effects.

Path	β	SE	Proportion Mediated	95% Bootstrap CI	
				Lower Limit	Upper Limit
Physical Activity → Character Strengths → Self-reported Academic Achievement	0.051	0.016	15.60%	0.022	0.085
Physical Activity → Self-Control → Self-reported Academic Achievement	0.039	0.014	11.93%	0.015	0.070
Physical Activity → Character Strengths → Self-Control → Self-reported Academic Achievement	0.027	0.008	8.26%	0.012	0.045
Total Indirect Effect	0.117	0.021	35.78%	0.079	0.160
Direct Effect	0.210	0.044	-	0.124	0.296
Total Effect	0.327	0.045	-	0.239	0.415

**Note:** all coefficients are standardized.

## Data Availability

The data supporting the findings of this study are available from the corresponding author upon reasonable request. The data are not publicly available due to privacy and ethical considerations.
